# Limited nucleotide pools restrict Epstein–Barr virus-mediated B-cell immortalization

**DOI:** 10.1038/oncsis.2017.46

**Published:** 2017-06-12

**Authors:** A Y Hafez, J E Messinger, K McFadden, G Fenyofalvi, C N Shepard, G M Lenzi, B Kim, M A Luftig

**Affiliations:** 1 Department of Molecular Genetics and Microbiology, Center for Virology, Duke University School of Medicine, Durham, NC, USA; 2 Department of Pediatrics, Center for Drug Discovery, School of Medicine, Emory University, Atlanta, GA, USA

## Abstract

Activation of cellular oncogenes as well as infection with tumor viruses can promote aberrant proliferation and activation of the host DNA damage response. Epstein–Barr virus (EBV) infection of primary human B cells induces a transient period of hyper-proliferation, but many of these infected cells succumb to an ataxia telangiectasia mutated/checkpoint kinase 2 (ATM/Chk2)-mediated senescence-like growth arrest. In this study, we assessed the role of DNA replicative stress and nucleotide pool levels in limiting EBV-infected B-cell outgrowth. We found that EBV triggered activation of the ataxia telangiectasia and Rad3-related (ATR) signaling pathway in the early rapidly proliferating cells, which were also significantly more sensitive to inhibition of the ATR pathway than late attenuated proliferating cells. Through nuclear halo assays, we determined that early EBV-infected cells displayed increased replicative stress and DNA damage relative to late proliferating cells. Finally, we found that early after infection, hyper-proliferating B cells exhibited limited deoxyribonucleotide triphosphate (dNTP) pools compared with late proliferating and EBV-immortalized lymphoblastoid cell lines with a specific loss of purine dNTPs. Importantly, supplementation with exogenous nucleosides before the period of hyper-proliferation markedly enhanced B-cell immortalization by EBV and rescued replicative stress. Together our results suggest that purine dNTP biosynthesis has a critical role in the early stages of EBV-mediated B-cell immortalization.

## Introduction

Aberrant cellular proliferation is first recognized by the DNA damage response (DDR), an innate tumor-suppressor pathway.^1, 2, 3, 4^ The activation of oncogenes by mutation or infection with an oncogenic virus triggers this response because of inappropriate entry into the cell cycle and unscheduled initiation of DNA replication. The DDR has thus come to be recognized as an important barrier to tumorigenesis.^1, 2, 5, 6, 7^ Unscheduled replication initiation induced by oncogene overexpression leads to exposed single-stranded DNA/double-stranded DNA junctions recognized by the ATR/Chk1 DDR signaling pathway, which can also be processed to double-stranded breaks recognized by the ATM/Chk2 pathway.^8, 9, 10^ Although normal levels of replicative stress experienced in every cell cycle leads to transient cell cycle arrest and DNA repair, the elevated DDR signaling observed following oncogene activation can promote apoptosis or senescence through signaling to the p53 pathway and other regulators of cell fate.^1, 6, 11, 12, 13^

Our model system for the study of innate tumor-suppressor responses is the infection of primary human B cells with the oncogenic herpesvirus Epstein–Barr virus (EBV). Although EBV latently infects nearly all adults worldwide, the virus causes B-cell lymphomas in immune suppressed individuals such as those following transplant or human immunodeficiency virus infection.^14, 15^ *In vitro*, EBV infection of primary human B cells leads to their transformation into indefinitely proliferating lymphoblastoid cell lines, or LCLs. However, only a small percentage of infected cells actually become LCLs suggesting that innate tumor-suppressor responses may restrict long-term outgrowth.^16, 17^ Indeed, our laboratory and others have found that the DDR is activated early after infection and the ATM/Chk2 pathway limits outgrowth through activation of a senescence-like growth arrest.^18, 19, 20, 21, 22^

Upon initial B-cell infection by EBV, the viral latent oncoproteins EBNA2 and EBNA-LP coordinate the upregulation of cellular gene expression that promotes cell cycle entry and rapid DNA replication and cell division.^23, 24, 25^ At 3 days post infection, EBV-infected B cells undergo a burst of proliferation in which cells divide up to three or four times within 24 h. Following this initial period of hyper-proliferation, the infected cells slow their proliferation rate to approximately one division per 24 h. The proliferative burst correlates with a period of activated DNA damage signaling. We found that the ATM/Chk2 pathway is activated and promotes a senescence-like growth arrest in these infected cells.^21^ Others have found that these cells also display elevated reactive oxygen species and telomere dysfunction that may induce ATM pathway activation.^19, 26, 27^ Early-infected cells also display aberrant karyotypes, but as infected cells grow out over a period of weeks into LCLs the DNA damage signaling wanes and these cells display stable karyotypes.^28^

Given the rapid proliferation of early EBV-infected cells, cellular DNA replicative stress during infection is a likely candidate for the upstream molecular source of the activated DNA damage observed in this system. Indeed, activation of the sensor of replicative stress, ATR, has been reported in early EBV-infected B cells.^20^ In other systems both *in vitro* and *in vivo*, the overexpression of oncogenes including c-Myc, H-ras^G12V^, cyclin E, and human papilloma virus E6 and E7 promotes replicative stress and ATR/Chk1 pathway activation.^29, 30, 31, 32^ Increased activity of this pathway triggers senescence while loss of one allele of ATR or Chk1 partially overrides senescence leading to increased genomic instability and tumorigenicity.^30^ However, at a higher level of ATR pathway inhibition the growth of tumors harboring oncogenic mutations can be suppressed with minimal impact on highly proliferative normal tissues, highlighting ATR inhibition as a promising therapeutic strategy.^33, 34, 35^ Inhibitors of this pathway are currently under development for a wide range of tumors that display high proliferative rates and increased markers of replicative stress.^36^

Recent studies linking the metabolic demands of oncogene-induced rapid cell proliferation to the DDR suggest that maintenance of deoxyribonucleotide triphosphate (dNTP) pools is critical to prevent DDR activation and oncogene-induced senescence.^7, 29, 31, 32, 37^ Specifically, HPV16 E6 and E7 expression in keratinocytes and H-Ras^G12V^ in fibroblasts leads to dNTP depletion and DNA replicative stress.^29, 37^ In both settings, providing exogenous nucleosides rescues replicative stress and overcomes oncogene-induced senescence. Therefore, regulation of dNTP pools is critical to support early steps in oncogenesis.

In this study, we examine the role of dNTP pools and replicative stress in regulating B-cell immortalization by EBV. Our prior work indicates that during the hyper-proliferative burst following EBV infection, cells that succumb to ATM/Chk2-mediated growth arrest have failed to upregulate oxidative phosphorylation and genes associated with mitochondrial biogenesis.^38^ We hypothesize that this metabolic imbalance may lead to a deficiency in dNTP levels necessary to sustain hyper-proliferation during the first rounds of B-cell division after infection. In this study, we directly measured DNA damage and replicative stress during early and late times after EBV infection of primary human B cells and assessed the role of the ATR/Chk1 signaling pathway in B-cell outgrowth. We also measured the levels of dNTPs during the course of infection and assessed the role of dNTP pools in B-cell immortalization by EBV.

## Results

### EBV infection of primary human B cells leads to increased ATR pathway activation early after infection

**Figure 1:**
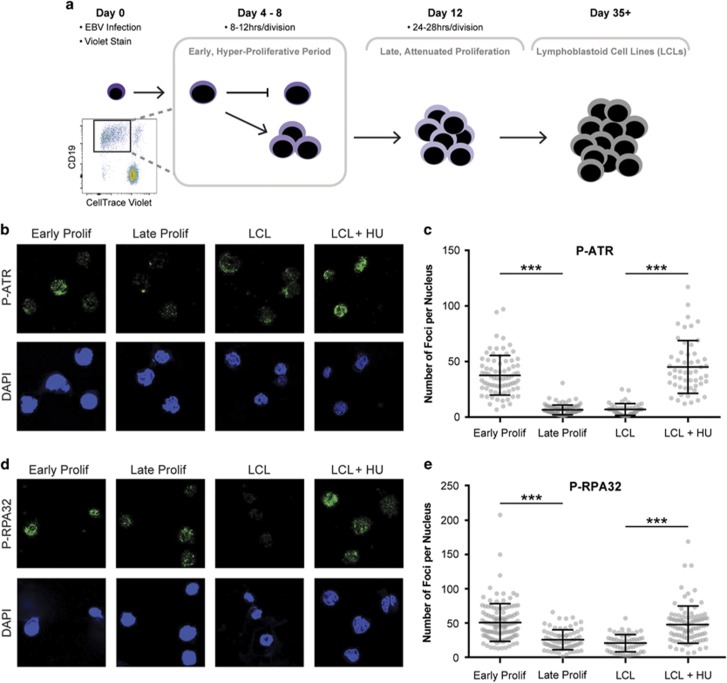
ATR pathway is activated in early, rapidly proliferating EBV-infected B cells and attenuated following hyper-proliferation. (**a**) Schematic demonstrating staining, infection and sorting protocol to separate early, hyper-proliferating populations (PA: proliferating, then arrested and PP: proliferating, then proliferating further), from the later, slower proliferating population, and then LCLs. (**b**) Immunofluorescence (IF) of phosphorylated ATR S428 (P-ATR) (green) and DAPI (blue) measured from sorted early and late proliferating B cells, untreated LCLs and 3 mM hydroxyurea (HU)-treated LCLs. (**c**) Number of P-ATR S428 foci per nucleus of sorted early and late proliferating B cells, untreated LCLs and 3 mM HU-treated LCLs. Error bars represent s.e.m. of three independent donors. ***P<0.001 as determined by a Mann–Whitney test. (**d**) IF of phosphorylated RPA32 S4/S8 (P-RPA32) (green) and DAPI (blue) measured from sorted early and late proliferating B cells, untreated LCLs, and 3 mM HU-treated LCLs. (**e**) Number of P-RPA32 S4/S8 foci per nucleus of sorted early and late proliferating B cells, untreated LCLs and 3 mM HU-treated LCLs. Error bars represent s.e.m. of three independent donors. ***P<0.001 as determined by a Mann–Whitney test.

We previously reported that upon EBV infection, B cells undergo a transient period of hyper-proliferation during which a cell can divide up to four times in 1 day.^21^ This increased division rate requires that infected cells replicate their genomes very rapidly, potentially leading to DNA replicative stress and activation of the ATR/Chk1 signaling pathway. To determine whether EBV induces ATR pathway activation, we analyzed infected B cells as they emerged from the resting state using a flow cytometry-based sorting approach and immunofluorescence for phosphorylated/activated ATR (P-ATR Ser428) and phosphorylated RPA32 (P-RPA Ser4/8). We first stained peripheral blood mononuclear cells (PBMCs) with a proliferation tracking dye, CellTrace Violet (CTV), and then infected with EBV at a multiplicity of infection such that every CD19^+^ B cell is latently infected with EBV.^21^ At 4 days post infection, we sorted the majority of infected PBMCs for CD19 positivity and dilution of CTV thereby isolating a purified population of infected, early rapidly proliferating B cells (Figure 1a). We allowed the remainder of the unsorted, infected cells to continue to proliferate for an additional 8 days and sorted again on CD19 and dilution of CTV, which had attenuated their proliferation rate as shown previously.^21^ We assayed these two cell populations for P-ATR and P-RPA32 by measuring the number of markers of replicative stress foci per nucleus. We found that early proliferating B cells exhibited significantly more activated P-ATR and P-RPA32 than late proliferating B cells (Figures 1b–e). We also found that EBV-immortalized LCLs displayed low levels of P-ATR and P-RPA32, similar to late proliferating cells, and LCLs induced to undergo replicative stress by treatment with hydroxyurea displayed significantly elevated levels of P-ATR and P-RPA32 foci (Figures 1b–e). These data suggest that EBV-induced hyper-proliferation triggers ATR pathway activation similar to that observed by others.^20^

### Early proliferating EBV-infected B cells are more sensitive to ATR and Chk1 pathway inhibition than late proliferating infected cells

**Figure 2:**
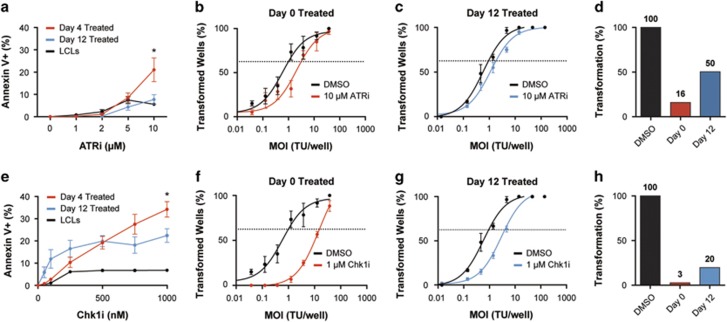
Early EBV-infected B cells are more sensitive to ATR and Chk1 inhibition than late proliferating B cells. (**a**) Percentage of Annexin V positive EBV-infected B cells treated with increasing concentration of ATR inhibitor, VE-821, on days 4 (red) or 12 (blue) post infection. Error bars represent s.e.m. of six independent donors and three LCLs. *P<0.05 as determined by Student’s *t*-test. (**b**) Quantification of EBV-induced B-cell outgrowth in the presence of either 0.1% DMSO (black) or 10 μM VE-821 (red) on day 0 post infection. The percentage of wells positive for LCL outgrowth 5 weeks post infection is shown relative to the transforming units (TU) of EBV B95.8 per well. Error bars represent s.e.m. of three independent donors. (**c**) Similar experiments were performed as in **b**, except that cells were treated with 10 μM VE-821 (red) on day 12 post infection. (**d**) Quantification of transformation efficiency from **b** and **c**. (**e**) Percentage of Annexin V positive EBV-infected B cells treated with increasing concentration of Chk1 inhibitor, CHIR-124, on days 4 (red) or 12 (blue) post infection. Error bars represent s.e.m. of six independent donors and three LCLs. *P<0.05 as determined by Student’s *t*-test. (**f**) Quantification of EBV-induced B-cell outgrowth in the presence of either 0.1% DMSO (black) or 1 μM CHIR-124 (red) on day 0 post infection. (**g**) Similar experiments were performed as in **f**, except that cells were treated with 1 μM CHIR-124 (red) on day 12 post infection. (**h**) Quantification of transformation efficiency from **f** and **g**.

Activation of the ATR signaling pathway in response to oncogene overexpression has been shown to provide a specific vulnerability for cell survival. Several groups have demonstrated that loss-of-function in the ATR/Chk1 pathway triggers apoptosis in cells with activated oncogene signaling or other inducers of replication stress.^33, 34, 35, 36^ To further investigate the role of the ATR pathway in EBV-infected B cells, we used a pharmacological approach targeting ATR and Chk1. First, we assessed the level of apoptosis induced by a selective ATR inhibitor (ATRi), VE-821,^39^ during the hyper-proliferative period early after infection. As indicated in Figure 2a, we observed a selective increase in apoptosis as measured by Annexin V positivity in cells undergoing hyper-proliferation following EBV infection (day 4) relative to those that had proceeded beyond the hyper-proliferative period (day 12) or LCLs (>day 35). As a complementary approach, we assessed the relative sensitivity of B cells to EBV transformation in the presence of ATRi at early and late times after infection. We observed that transformation was more potently inhibited by ATRi when administered at early times post infection (day 0) as compared with later times (day 12) (Figures 2b–d). These data collectively suggest that EBV-infected B cells undergoing hyper-proliferation, where ATR is activated because of replicative stress, are also hyper-sensitive to ATR inhibition and depend on ATR for their survival.

To corroborate these findings and assess the role of the downstream ATR effector Chk1, we selectively inhibited this kinase using CHIR-124.^^40^^ Consistent with the ATR inhibition results, we observed that EBV-infected cells treated before hyper-proliferation were more sensitive to Chk1 inhibition than those treated following the hyper-proliferative period or LCLs (Figure 2e). Furthermore, inhibition of Chk1 during the hyper-proliferative period markedly suppressed EBV transformation, whereas treatment at later times during infection had a less pronounced effect (Figures 2f–h). Thus, we conclude that ATR and Chk1 protect early proliferating EBV-infected B cells from apoptosis during the hyper-proliferative period and therefore are critical for long-term outgrowth into LCLs. This ATR and Chk1 protective function is consistent with reports from the replicative stress field.^41^

### Early proliferating EBV-infected B cells experience DNA damage and replication stress, which is resolved later in the immortalization process

Here we have reported that early proliferating EBV-infected B cells activate the ATR/Chk1 replicative stress pathway and require this pathway for survival. However, several groups have demonstrated DDR pathway activation in the absence of overt signs of DNA damage.^42, 43^ Therefore, we sought to directly measure the presence of DNA damage and replicative stress in early EBV-infected primary B cells.

**Figure 3:**
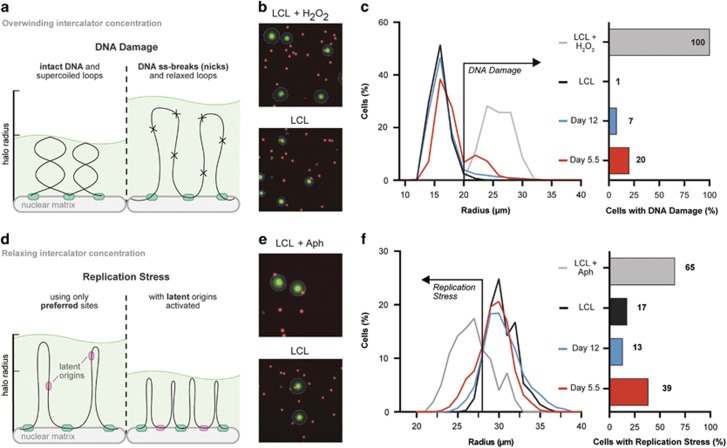
EBV-infected B cells experience DNA damage and replicative stress early after infection. (**a**) Schematic of fluid halo assay investigating DNA damage. (**b**) Representative images of control LCLs that were untreated or treated with hydrogen peroxide to induce DNA damage and subsequently increase halo size. Blue outline indicates halo boundaries as detected by Cellomics ArrayScan. (**c**) Histogram showing distribution of halo sizes of sorted early (day 5.5) (red) and late proliferating B cells (day 12) (blue), untreated LCLs (black), and hydrogen peroxide-treated LCLs (gray). Cells experiencing DNA damage were gated such that all radii greater than untreated LCLs were considered to be experiencing DNA damage. (**d**) Schematic of fluid halo assay investigating replicative stress. (**e**) Representative images of control LCLs that were untreated or treated with aphidicolin to induce replicative stress and subsequently reduce halo size. Blue outline indicates halo boundaries as detected by Cellomics ArrayScan. (**f**) Histogram showing distribution of halo sizes of sorted early (day 5.5) (red) and late proliferating B cells (day 12) (blue), untreated LCLs (black), and aphidicolin-treated LCLs (gray). Cells experiencing replicative stress were gated such that all radii smaller than untreated LCLs were considered to be undergoing replicative stress.

To assess DNA damage, we conducted a fluid halo assay, which is capable of detecting DNA single-stranded breaks or nicks.^44, 45^ EBV-infected, proliferating B cells were sorted early or late after infection. These cells were gently lysed to release the nuclei. Nuclei were de-chromatinized by high salt solution and the resulting nuclear halos, consisting of supercoiled DNA loops periodically attached to the central nuclear matrix, were stained with an over-winding concentration of the DNA intercalating agent, SYBR gold (10X) (schematic in Figure 3a). As an over-winding concentration was used, cells experiencing DNA damage will have longer chromatin loops as evidenced by larger nuclear halos, whereas undamaged DNA would remain tightly compacted and those nuclei would have smaller halos.^44, 45^ Consistently, control LCLs treated with hydrogen peroxide displayed larger halo sizes than untreated LCLs when halo assays were performed using over-winding concentrations (Figures 3b and c). We observed that a greater percentage of early proliferating EBV-infected B cells displayed DNA damage as compared with late proliferating cells and LCLs (Figure 3c).

Next, we investigated the presence of replicative stress as a cause of this DNA damage. When replicative stress occurs, latent origins of replication can fire to help compensate for increased DNA replication demands.^46^ To detect changes in replication, we performed fluid halo assays, but at the relaxation concentration of SYBR Gold (0.95X) such that latent origins associating with the nuclear matrix generate smaller nuclear halos (Figure 3d).^47^ Firing of the dormant origins help compensate for the decreased replication speed or increased DNA replication demands.^46^ Consistently, triggering of replicative stress by inhibiting DNA replication in LCLs with aphidicolin led to substantially decreased halo sizes relative to untreated LCLs (Figures 3e and f). When we assayed early and later proliferating EBV-infected cells, we found that the early hyper-proliferating cells displayed greater levels of replicative stress than those at later times post infection (day 12 or LCLs) (Figures 3e and f). Taken together, these data suggest that replicative stress occurs in early proliferating EBV-infected B cells, which may lead to the observed DNA damage that will be repaired later in those cells that continue to proliferate past the period of hyper-proliferation.

### Limited dNTP pools in early proliferating B cells suppresses EBV-mediated transformation

**Figure 4:**
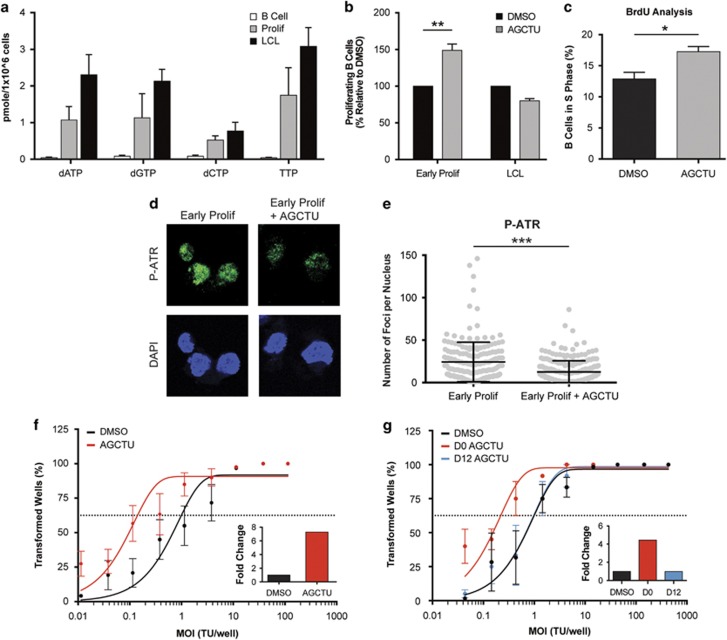
Nucleoside supplementation rescues EBV-induced growth arrest and presence of replicative stress. (**a**) Quantification of individual deoxyribonucleotide pools, including dATP, dGTP, dCTP and TTP measured from sorted uninfected B cells (white), infected early proliferating B cells (gray) and LCLs (black). (**b**) Percentage of proliferating CD19^+^ B cells was determined for early proliferating infected B cells and LCLs that were treated with DMSO (black) or supplemented with 30 μM nucleosides (AGCTU) (gray) at the time of infection. The data were analyzed by FACS at day 14 post infection. Error bars represent s.e.m. of three independent donors. **P<0.01 as determined by a Student’s *t*-test. (**c**) Percentage of B cells in S phase that were treated with DMSO (black) and supplemented with 30 μM nucleosides (AGCTU) (gray) at time of infection. The BrdU cell cycle profiles were analyzed by FACS on day 6 post infection. Error bars represent s.e.m. of three independent donors. *P<0.05 as determined by a Student’s *t*-test. (**d**) IF of P-ATR (green) and DAPI (blue) measured from early proliferating B cells mock-treated with DMSO (Early Prolif) and supplemented with 30 μM nucleosides on day 0 (Early Prolif+AGCTU). (**e**) Number of P-ATR S428 foci per nucleus of sorted early proliferating B cells mock-treated with DMSO (Early Prolif) and 30 μM nucleosides (AGCTU) supplemented on day 0 (Early Prolif+AGCTU). (**f**) Quantification of EBV-infected B-cell outgrowth following PBMC infection in the presence of DMSO (black) or 30 μM nucleosides (AGCTU) (red) at time of infection. The percentages of wells positive for LCLs at 5 weeks post infection are plotted relative to the transforming units (TU) of B95-8 virus per well. Error bars represent s.e.m. Dotted line represents 62.5% positive wells, which indicates outgrowth from the virus amount in the *x* axis of a single LCL per well based on a Poisson’s distribution. (**f**, inset) Fold change of the transformation efficiency. (**g**) Similar experiments were performed as in **f**, except treating with DMSO (black) at the time of infection, 30 μM nucleosides (AGCTU) (red) at the time of infection (red) and day 12 post infection (gray). (**g**, inset) Fold change of the transformation efficiency.

The presence of DNA damage and replicative stress in EBV-infected early, rapidly proliferating B cells suggests that nucleotide pools may be limiting in these cells. Therefore, we sought to measure the levels of individual dNTPs following EBV infection of primary B cells, during early proliferation, and through LCL outgrowth. We found that early proliferating B cells contained much higher levels of dNTPs relative to resting B cells, as expected. However, in the transition from early proliferation through LCL outgrowth, the dNTP levels were further increased, particularly for the purine dNTPs (Figure 4a).

We next sought to determine whether this relative limitation in dNTPs during early proliferation may functionally impede the outgrowth of EBV-immortalized cells. We supplemented the B-cell growth media with adenosine, guanosine, cytosine, uridine and thymidine (AGCTU) concurrent with EBV infection and this led to an increase in the number of CD19^+^ proliferating B cells at day 14 post infection relative to untreated cells (Figure 4b). However, supplementation of LCLs with AGCTU nucleosides had no effect on B-cell proliferation (Figure 4b). Furthermore, we observed that nucleoside supplementation overcame a previously defined G1/S phase arrest that occurs before OIS in these early-infected cells (Figure 4c and McFadden *et al.*^38^). Importantly, supplementation with nucleosides rescued replicative stress as observed by a decrease in P-ATR replicative stress foci (Figures 4d and e).

To determine if low levels of dNTP pools contributed to the restriction of EBV-mediated long-term outgrowth, we simultaneously infected PBMCs with EBV and supplemented the growth media with AGCTU nucleosides. We observed a significant increase in EBV-mediated transformation efficiency with supplementation of nucleosides relative to the dimethylsulfoxide (DMSO)-treated infected PBMCs (Figure 4f). We next assessed whether the time of addition of nucleosides was important in regulating transformation efficiency given our findings of elevated replicative stress markers only during the early hyper-proliferative phase of latent infection. Nucleosides supplemented concurrent with infection markedly increased transformation efficiency of EBV-infected B cells; however, addition of nucleosides on day 12 post infection, after the hyper-proliferative period, had no effect on transformation efficiency (Figure 4g). Collectively, these findings suggest that limited nucleotide pools contribute to replicative stress and arrest of early proliferating B cells, ultimately suppressing EBV-mediated transformation.

### Purine dNTP pools are reduced in early proliferating EBV-infected B cells and supplementation with purine nucleosides alone rescues viral-mediated transformation

**Figure 5:**
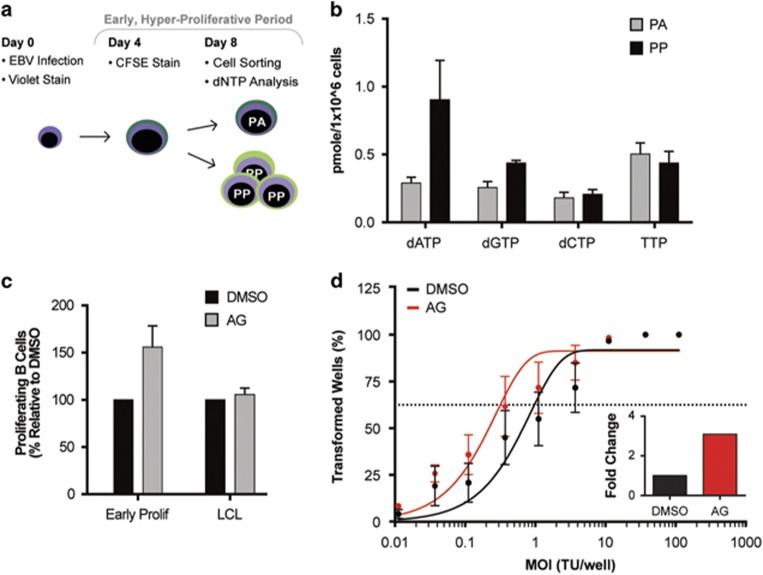
Supplementation with purine nucleosides alone rescues EBV-induced growth arrest. (**a**) Schematic demonstrating staining, infection and sorting protocol to separate proliferating, then arrested (PA) from proliferating, then proliferating further (PP) cells within the early proliferating period. (**b**) Quantification of individual deoxyribonucleotide pools, including dATP, dGTP, dCTP and TTP measured from sorted PA cells (gray) and PP cells (black). (**c**) Percentage of proliferating CD19^+^ B cells was determined for early proliferating infected B cells and LCLs that were treated with DMSO (black) or supplemented with 30 μM purine nucleosides (AG) (gray) at the time of infection. The data were analyzed by FACS at day 14 post infection. Error bars represent s.e.m. of three independent donors. (**d**) Quantification of EBV-infected B-cell outgrowth following PBMC infection in the presence of DMSO (black) or 30 μM purine nucleosides (AG) (red) at time of infection similar to experiments performed in Figure 4f and g. (**d**, inset) Fold change of the transformation efficiency.

To further investigate the effect of nucleotide pool depletion on arrest of EBV-infected B cells, we specifically measured individual dNTP pools of arrested B cells. Using a previously established double-staining technique to track proliferation of cells using CTV and 6-carboxyfluorescein succinimidyl ester, we were able to separate the EBV-infected B cells that proliferate and then arrest (PA) early after infection compared with the proliferating B cells that continue to proliferate (PP)^38^ (Figure 5a). We measured dNTP levels in these populations and found that the PA population exhibited lower levels of purine dNTP pools (dATP and dGTP) compared with the PP population (Figure 5b).

We next wanted to determine whether limited purine dNTP pools, specifically, influenced cellular arrest and suppression of EBV-mediated transformation. We supplemented the B-cell growth media with only adenosine and guanosine ribonucleosides (AG) on day 0 post infection and conducted fluorescence-activated cell sorting (FACS) on day 14 post infection to analyze early proliferation of CD19^+^ B cells. Early proliferating B cells exhibited a similar increase in B-cell proliferation with supplementation of purine nucleosides as that seen in Figure 4b with AGCTU nucleoside addition (Figure 5c). Similar to that observed with AGCTU, LCL proliferation was unaffected by AG supplementation (Figure 5c). To gain a functional understanding of the role of purine nucleosides in long-term EBV outgrowth, we conducted transformation assays by supplementing the growth media with purine nucleosides. Purine nucleosides supplemented on day 0 post infection increased transformation efficiency of EBV-infected B cells by three-fold over DMSO-treated infected cells (Figure 5d). Together these findings suggest that purine nucleotide pools are a uniquely limiting factor for EBV-mediated hyper-proliferation and transformation.

## Discussion

The recognition of oncogene-mediated aberrant proliferation by the DDR signaling pathway is among the earliest innate tumor-suppressor responses. When EBV infects primary B cells, it must drive cell proliferation to establish a reservoir of latently infected cells. We have previously found that EBV induces rapid proliferation such that at approximately 3 days post infection the first three to four cell divisions occur within a 24-h period.^21^ The majority of infected cells that begin this hyper-proliferation program ultimately succumb to an ATM/Chk2-dependent senescence-like growth arrest.^21, 38^ In this study, we sought to determine the upstream molecular source of the DDR. We found that early EBV-infected hyper-proliferating cells display evidence of DNA damage and replicative stress using fluid halo assays and assays for ATR/Chk1 pathway activation. We further observed reduced dNTPs, particularly purine dNTPs, in early-infected cells that led to their inefficient outgrowth. Supplementation of nucleosides rescued activation of replicative stress markers and cellular arrest. Furthermore, supplementation of specifically purine nucleosides at early times post infection facilitated EBV-mediated B-cell outgrowth suggesting that purine biosynthesis is a major limiting step in EBV transformation.

Depletion of dNTP pools has been linked to induction of replicative stress. We demonstrated that early, hyper-proliferating EBV-infected cells exhibit increased replicative stress by nuclear halo assays. Although depleted dNTPs can generate replication fork collapse randomly throughout the genome, these lesions are typically efficiently repaired.^48^ In contrast, replicative stress at genomic sites that are difficult to repair often lead to persistent DNA damage signaling and senescence or apoptosis.^49^ A primary site of irreparable DNA damage important for triggering senescence is telomeres.^50^ Indeed, the Herbig laboratory has demonstrated that oncogene-induced senescence is mediated by replicative stress and irreparable DDR signaling at telomeres.^51^ Masucci and colleagues have previously demonstrated that early EBV-infected cells display evidence of telomere-associated DNA damage.^19^ Our prior work on ATM-mediated growth suppression of early EBV-infected cells together with our demonstration here of replicative stress and telomere dysfunction by the Masucci group is therefore consistent with a model whereby replicative stress at telomeres is the key molecular source of persistent DNA damage triggering senescence in early-infected cells.

An interesting, emerging consequence of hyper-proliferation and replicative stress in pre-neoplastic and neoplastic tissue is a vulnerability to ATR and Chk1 pathway inhibition relative to cells with normal proliferation rates.^33, 34, 35^ For example, cells expressing oncogenic Ras display substantially increased genomic instability and cell death when ATR levels are depleted genetically or pharmacologically.^30^ Similarly, amplification of Myc or cyclin E, commonly found in many cancers, leads to heightened sensitivity to ATR inhibition.^35^ In our studies, we find that early EBV-infected, rapidly proliferating cells are more sensitive to ATR and Chk1 inhibition than later, normally proliferating EBV-infected cells. Although another group has recently published that ATR-Chk1 pathway facilitates EBV-mediated transformation of tonsillar B cells,^52^ we have examined this further in a more rigorous manner by sorting very specific early proliferating EBV-infected B cells toward characterizing the hyper-sensitivity of these cells relative to later proliferating B cells and LCLs. Furthermore, prior work has found that early EBV-infected cells display activated ATR; this work has shown that Chk1 was not phosphorylated on Ser 345.^20^ As cross-talk among downstream phosphorylation targets is common in DDR signaling pathways,^53, 54^ we hypothesize that Chk1 is phosphorylated on Ser 317 or other sites that may trigger cell cycle arrest downstream of ATR in EBV-induced replicative stress. Ultimately, the DNA damage recognized by the ATR/Chk1 pathway following reduced dNTP pools and replicative stress must be reconciled during early EBV infection to promote the efficient outgrowth of immortalized LCLs.

In summary, EBV infection of primary B cells initially transits through a period of rapid proliferation presenting a high demand for nucleotide synthesis. Viral latency transcription factors must therefore promote activity of E2F complexes to enhance cell cycle progression. The consequences of hyper-replication during these early rapid rounds of proliferation is replicative stress and activation of the DDR. The ATR/Chk1 pathway is initially activated and is important for B-cell outgrowth. However, failure to repair damaged DNA at key sites, such as telomeres, results in a persistent ATM/Chk2-mediated DDR that triggers senescence. Infected cells that overcome this initial challenge to B-cell hyper-proliferation ultimately grow out as LCLs *in vitro*. *In vivo*, T-cell pressure against latent infection pushes these infected cells into true latency where no viral proteins are expressed.^55^ However, in immune suppressed individuals these continuously growing latently infected cells are the precursors to B-cell lymphomas. In the future, it will be important to discern whether EBV-positive lymphomas *in vivo* display hallmarks of overcoming an initial replicative stress mediated tumor-suppressive DDR.

## Materials and methods

### Viruses and cells

B95-8 virus was produced from the B95-8 Z-HT cell line as previously described.^56^ Buffy coats were obtained from normal donors through the Gulf Coast Regional Blood Center and PBMCs were isolated by Ficoll Histopaque-1077 gradient (Sigma, St Louis, MO, USA; #H8889). Primary cells were cultured in RPMI-1640 with 15% fetal bovine serum, 2 mM l-glutamine, penicillin and streptomycin (1X, Sigma; #G6784) (R15) and 0.5 μg/ml Cyclosporin A (Sigma; #30024). All bulk infections were performed by incubating cells with B95-8 Z-HT supernatants (1 ml per 10^6^ B cells calculated from within PBMC population) for 1 h at 37 °C in a CO_2_ incubator followed by washing in phosphate-buffered saline and resuspending in R15 media+Cyclosporin A. Typical bulk infections were done on 5 × 10^8^ PBMCs. LCLs were generated from normal donors by continuous growth of EBV-infected primary B cells for greater than two months. LCLs were cultured in RPMI with 10% fetal bovine serum (R10).

### Chemicals

Hydroxyurea (Sigma; #H8627) was resuspended directly in R15 media at 3 mM. Adenosine (Sigma; #A9251), cytosine (Sigma; #C3506), thymidine (Sigma; #T9250) and uridine (Sigma; #U3750) were resuspended at 3 mM in UltraPure distilled water (Invitrogen, Carlsbad, CA, USA; #10977-015). Guanosine (Sigma; #G6752) was resuspended at 30 mM in DMSO. Both VE-821 and CHIR-124 (Selleckchem, Boston, MA, USA; #S8007 and #S2683, respectively) were resuspended in DMSO at 10 mM.

### Antibodies

Mouse anti-human CD19 antibody (clone 33-6-6, kind gift of Dr Tom Tedder) conjugated with either allophycocyanin (APC) or phycoerythrin (PE) was used as a surface B-cell marker in flow cytometry. Mouse anti-human CD19-phycoerythrin-Cyanine7 antibody (eBioscience, San Diego, CA, USA; #25-0199-42) was used as an additional B-cell surface marker. All surface B-cell markers were used at 1μl per 10^6^ cells. Phosphorylated ATR (S428) (Santa Cruz Biotechnology, Dallas, TX, USA; #sc-109912) and Phosphorylated RPA32 (S4/S8) (Bethyl Laboratories, Montgomery, TX, USA; #A300-245A) were used as markers of replicative stress for immunofluorescence at 1:50 and 1:500, respectively.

### Infections and cell sorting

PBMCs were isolated from a buffy coat and stained with CTV using the manufacturer’s suggested protocol (Invitrogen; #C34557) followed by infection with EBV at a multiplicity of infection (MOI) of 5 (such that all infected B cells are positive for EBNA-LP). Proliferation was monitored in CD19^+^ B cells by the dilution of the CTV stain for up to 14 days post infection on a BD FACS Canto II (Becton Dickinson, Franklin Lakes, NJ, USA) and analyzed using FlowJo 10.0 software (TreeStar) (FlowJo, Ashland, OR, USA). CD19-positive cells were sorted into early and late population doublings based upon their CTV profile using either a Beckman Coulter Astrios or Beckman Coulter MoFlo XDP sorter (Beckman Coulter, Brea, CA, USA). Sorting to capture early proliferating and late proliferating populations were conducted as follows:

#### Immunofluorescence analysis

Infected B cells were sorted such that cells were isolated that corresponded to populations of either 1–2 divisions or >5 divisions as determined by CTV profile on days 4 and 12, respectively.

#### Fluid halo assays

Cell populations that doubled 1–2 times were sorted on day 5.5 and populations that doubled over five times were sorted on day 12 for analysis by Fluid Halo assay. Populations positive for propidium iodide were gated out to remove dead cells.

#### dNTP analysis

Infected cell populations were sorted on day 8 for proliferating B cells that divided over five times. Alternatively, to specifically capture early proliferating and arresting B cells infected PBMCs were stained with CTV (Invitrogen; #C34557) on day 0 post infection. The cells were cultured in R15 media for 4 days before staining with 6-carboxyfluorescein succinimidyl ester (Sigma; #21888). The samples were resuspended in fresh R15 media and cells were sorted into arrested and proliferating populations on day 8 based on both the CTV and 6-carboxyfluorescein succinimidyl ester fluorescence profile.

### Immunofluorescence

Immunofluorescence was performed as previously published.^38^

### Fluid halo assay

Cells were harvested and resuspended with 10^6^ cells/ml in an isotonic, low ionic strength lysis buffer containing 300 mM sucrose, 5 mM EDTA, 1 mM EGTA, 20 mM Tris pH 7.0, 1 mM spermine and Triton X-100 0.1% (w/w) on ice for 7 min. The lysate was diluted 100 × with the above isotonic buffer without Triton × -100 and infused with allophycocyanin CaliBRITE beads (BD Biosciences, Franklin Lakes, NJ, USA; #340386). Cells were transferred in 100 μl aliquots into the wells of alpha-poly-L-lysine (MW 150 000–300 000) (Sigma; #P1399) coated 96-well plates (Corning, Corning, NY, USA; #3904) and centrifuged at 1000 × *g* for 10 min at 4 °C. Halos were generated by removing 70 μl from the supernatant and diluting each well 1:10 with nuclear extraction buffer containing 20 mM Tris pH 7.5, 2.22 m NaCl, 1 mM EGTA and 5 mM EDTA with either an over-winding (10 ×) or relaxation (0.95 ×) final concentration of SYBR Gold (Thermo Fisher Scientific, Waltham, MA, USA; #S11494). Plates were then sealed and analyzed by Cellomics ArrayScan V^TI^ analyzer (ThermoFisher Scientific, Waltham, MA, USA) for nuclear halo size. Control LCLs were treated with either aphidicolin (1 μM, 48 h) (Sigma; A0871), which was dissolved in DMSO and stored at −80 °C as a 1 mg/ml or hydrogen peroxide (100 μM, 15 min, RT) (Sigma; #216763), which was stored at 4 °C as a 30% wt stock solution (100 000 ×) in sterile H_2_O to induce maximal replicative stress or DNA damage, respectively. Cells experiencing replicative stress were determined to have smaller halo sizes than LCLs at the relaxation SYBR Gold concentration, whereas cells with DNA damage were determined to have larger halo sizes than LCLs at the overwinding intercalator concentration. Only G1 phase cells were included in the analysis. Doublets and larger clusters were gated out from the analysis based on object shape, whereas cell debris was excluded by object intensity. To prevent the inclusion of halos in the analysis that could have been damaged by the light source of the scanning microscope, only the first nine vision fields were included in the analysis from each well.

### Apoptosis assays

PBMCs were isolated, stained with CTV and infected with EBV B95-8 as previously described. On days 4 or 12, cells were plated into 24-well plates and treated with 0.1% DMSO or 1, 10, 50, 100, 1000, 2000, 5000 or 10 000 nM VE-821. For the CHIR-124 assays, cells were treated either with 0.1% DMSO or 50, 100, 250, 500, 750 or 1000 nM CHIR-124. Two days post treatment, cells were stained with CD19-phycoerythrin and Annexin V-allophycocyanin (eBioscience; #17-8007-74) or Annexin V-FITC (Biolegend, San Diego, CA, USA; #640906) for 20 min at 4 °C before FACS analysis. Proliferating cells were determined by CTV profile and this population was gated to determine Annexin V-positive cells. All FACS data were analyzed using FlowJo 10.0 software (FlowJo, Ashland, OR, USA).

### Nucleotide pool measurement

dNTP extraction and measurement was conducted as previously published.^57^

### Cell cycle analysis

Isolated PBMCs were infected with EBV B95-8 and supplemented with 0.1% DMSO, 30 μM AGCTU or 30 μM AG on day 0 post infection. BrdU cell cycle analysis was conducted on day 6 post infection using BD Pharmingen allophycocyanin Flow Kit (Becton Dickinson, Franklin Lakes, NJ, USA; #552598) and analyzed using FlowJo 10.0 software.

### Transformation assay

EBV B95-8 infection of human PBMCs was performed in the presence of 0.1% DMSO, 30 μM AGCTU, 30 μM AG, 1 μM CHIR-124 or 10 μM VE-821 added at varying times post infection. B95-8 Z-HT supernatant was titrated from 300 μl/10^7^ PBMCs to 0.03 μl/10^7^ PBMCs. In all, 7 × 10^6^ infected PBMCs were seeded in 20 wells of a 96-well plate for each infection point. The percentage of wells positive for B-cell outgrowth (LCL) at 5 weeks post infection was plotted relative to the multiplicity of infection per well. The efficiency of transformation was determined as published where the amount of B95-8 virus necessary to yield 62.5% of positive wells was considered 1 transforming unit per well.
