# Supplementary material for: Limited nucleotide pools restrict Epstein–Barr virus-mediated B-cell immortalization
Source: Oncogenesis. 2017 Jun 12;6(6):e349–. doi: 10.1038/oncsis.2017.46 (PMC5519195; doi:10.1038/oncsis.2017.46)
Supplement: Supplementary Methods [file oncsis201746x1.docx]

**Supplementary methods**

**IF protocol.** EBV B95-8-infected B cells were pelleted, resuspended in 25μl of PBS, spread on a microscope slide and dried at 37°C for 15 minutes. Cells were fixed in 4% paraformaldehyde for 15 minutes at 4°C, washed in PBS, permeablized in PBS containing 0.2% Triton X-100 for 10 minutes and then blocked in PBS with 0.2% Triton X-100 containing 5% normal goat serum for 1 hour. Primary antibodies were incubated overnight at 4°C followed by secondary antibody incubation with AlexaFluor 488 goat anti-rabbit IgG (Life Technologies, Carlsbad, CA, USA #A11034) for 2 hours. Slides were mounted in Vectashield containing DAPI (Vector Laboratories, Burlingame, CA, USA #H-1200). All immunofluorescence slides were visualized using Zeiss 780 upright confocal microscope and images were analyzed using ImageJ version 2.0.

**Fluid Halo Assay.** Cells were harvested and resuspended with 10^6^ cells/mL in an isotonic, low ionic strength lysis buffer containing 300 mM sucrose, 20mM Tris pH 7.0, 1 mM spermine, and Triton X-100 0.1% (w/w) on ice for 7 min and infused with APC calibrate beads (BD Biosciences, Franklin Lakes, NJ, USA 340386). Cells were transferred in 100 μL aliquots into the wells of alpha-poly-L-lysine (MW 150 000-300 000, Sigma) coated 96 well plates and centrifuged at 1000xg for 10 min at 4°C. Halos were generated by diluting each well 1:1 with nuclear extraction buffer containing 20 mM Tris pH 7.5, 2.22 M NaCl, 1 mM EGTA, and 5 mM EDTA with either an over-winding (10x) or relaxation (0.95x) concentration of SYBR Gold (Thermo Fisher Scientific, Waltham, MA, USA #S11494) Plates were then sealed and analyzed by Cellomics ArrayScan V^TI^ analyzer for nuclear halo size. Control LCLs were treated with either aphidicolin (Sigma, A0871), which was dissolved in DMSO and stored at 1 mg/mL or hydrogen peroxide (Sigma, #216763) which was stored as a 30% wt stock solution (100 000x) in sterile H_2_O to induce replicative stress or DNA damage, respectively. Cells experiencing replicative stress were determined to have smaller halo sizes than LCLs. Cells experiencing DNA damage were determined to have larger halo sizes than LCLs. Doublets and larger clusters were eliminated from the analysis based on object shape, whereas cell debris was excluded by object intensity. To prevent the inclusion of halos in the analysis that could have been damaged by the light source of the scanning microscope, only the first nine vision fields were included in the analysis from each well.

**Infections and Cell Sorting.** Sorting to capture early proliferating and late proliferating populations were conducted as follows:

*Immunofluorescence analysis*:

Infected B cells were sorted such that cells were isolated that corresponded to populations of either 1-2 divisions or greater than 5 divisions as determined by CellTrace Violet profile on day 4 and 12, respectively.

*Fluid Halo assays*:

Cell populations that doubled 1-2 times were sorted on day 5.5 and populations that doubled over 5 times were sorted on day 12 for analysis by Fluid Halo assay. Populations positive for propidium iodide were gated out to remove dead cells.

*dNTP analysis*:

Infected cell populations were sorted on day 8 for proliferating B cells that divided over 5 times. Alternatively, to specifically capture early proliferating and arresting B cells infected PBMCs were stained with CellTrace Violet (Invitrogen, #C34557) on Day 0 post infection. The cells were cultured in R15 media with or without the presence of 30 μM nucleosides for 4 days prior to staining with 6-carboxyfluorescein succinimidyl ester (CFSE, Sigma, #21888). The samples were resuspended in fresh R15 media with or without the presence of 30 μM nucleosides and cells were sorted into arrested and proliferating populations on day 8 based on both the CellTrace Violet and CFSE fluorescence profile.
